# The “common soil” theory: coronary disease, diabetes and inflammation

**DOI:** 10.1590/S1516-31802005000500001

**Published:** 2005-09-01

“*Type 2 (non-insulin-dependent) diabetes mellitus and coronary heart disease: chicken, egg, or neither?*”: this was the question posed by Jarrett in 1984, on the basis of the knowledge available at that time that the risk of coronary disease was greater among individuals who had recently become diabetic than among those whose disease had been known of and diagnosed for a longer time.^1^ In 1995, Stern put forward the idea that diabetes and coronary disease both came from a “common soil”.^2^ The basis for this was the early environment theory for both diabetes and coronary disease, which started to gain strength and publicity through the publications of Barker et al., who described how, in the United Kingdom, regions that had presented high rates of infant mortality 50 years earlier were the ones with greater prevalence of diabetes and incidence of coronary disease decades later.^3^ Subsequently, they showed that inadequate nutritional conditions during the perinatal period were associated with increased incidence of hypertension, diabetes and coronary disease.^4–6^ Barker et al. put forward the hypothesis that fetal malnutrition could lead to some type of misregulation in pancreatic beta cells and, at the same time, malnutrition would give rise to fewer adipocytes, which in a subsequent situation with an excess of calorie offer would tend to cause hypertrophy in these cells, with undesired metabolic consequences.^3^

During this same period, the description by Reaven of the “X syndrome” also gained form and publicity. This subsequently became known as insulin resistance syndrome.^7^ The creation of this theory placed abdominal obesity as the basis for concomitant appearance of arterial hypertension, glucose intolerance and dyslipidemia (later on, this would be the combination of high triglycerides and lowering of the high-density lipoprotein fraction of cholesterol). Such observations are believed to have been started in France in the 1940s, by Jean Vague in the periodical La Presse Medicale in 1947 (vol. 55, p. 339-40) and 1949 (vol. 57, p. 556-7 e 835-7). These were published in French, and their first presentation in English, in 1956, did not receive the attention they were due.^8^ The first publications to associate abdominal obesity with diabetes and coronary disease came from the results of a 17-year follow-up of a cohort of men born in Gothenburg (Sweden) in 1913. These studies showed that regional adiposity presented a greater causal relationship with both coronary disease and with diabetes than did the body mass index^9,10^

In the 1990s, other findings showed that the intersection between diabetes and coronary disease went a bit beyond a mere cause-effect relationship such as the one that exists between the smoking habit and the incidence of lung cancer, for example. One interesting development was that the counter-intuitive effect of moderate use of alcoholic drinks as a protective factor against angina pectoris and myocardial infarction^11^ was also described in relation to the incidence of diabetes.^12^ Along the same lines, the moderate use of alcoholic drinks by diabetics reduced their risk that coronary diseases might appear to a similar extent as observed among non-diabetics who drank regularly.^13,14^ Another relevant description that is still controversial came from a study carried out in Finland, which showed that the mortality due to coronary disease had similar rates among participants with diabetes but without coronary disease and among those with coronary disease but without diabetes.^15^ These findings were not repeated in other cohorts studied, but the impact of the length of time with diabetes greatly collaborated towards bringing together the risk of death due to coronary disease between diabetics without previous history of coronary disease and individuals with the disease but without diabetes.^16–18^ However, there is no divergence with regard to the fact that an association between previous diagnoses of diabetes and coronary disease increases the risk of death tenfold, in comparison with individuals without such conditions. One effect on the demographic indicators may be the slowing down of the fall in mortality due to coronary disease due to the impact of diabetes and obesity, a situation that has been described in the United States and also in the metropolitan regions of Brazil.^19,20^

Since the end of the 1980s, the comprehension that regional adiposity is an independent risk factor of great importance with regard to the appearance of both diabetes and coronary heart disease has consolidated in such a way that a new disease, the “metabolic syndrome”, has become incorporated into medical jargon in substitution for the old “X syndrome”. In addition to the states already cited (abdominal obesity, dyslipidemia, hypertension and glucose intolerance), pro-thrombolytic and pro-inflammatory states also appear in the new definition.^21^ Subsequent to this, a new and fascinating field of research opened up that was associated both with diabetes and with coronary disease: the inflammatory process that underlies and perhaps is at the origin of both processes.^22–26^

Considering the impact of obesity and diabetes on our environment, studies focusing on the inflammatory process in the genesis of both these diseases will be very welcome.
